# Genotypic and Allelic Distribution of the *CD36* rs1761667 Polymorphism in High-Level Moroccan Athletes: A Pilot Study

**DOI:** 10.3390/genes15040419

**Published:** 2024-03-27

**Authors:** El Mokhtar El Ouali, Jihan Kartibou, Juan Del Coso, Badreddine El Makhzen, Laila Bouguenouch, Sanae El Harane, Bouchra Taib, Katja Weiss, Beat Knechtle, Abdelhalem Mesfioui, Hassane Zouhal

**Affiliations:** 1 Laboratory of Biology and Health, Department of Biology, Ibn Tofail University, Kenitra 14000, Morocco; elmokhtar.elouali@uit.ac.ma (E.M.E.O.); j.kartibou@gmail.com (J.K.); abdelhalem.mesfioui@gmail.com (A.M.); 2 Sport Sciences Research Centre, Rey Juan Carlos University, 28943 Fuenlabrada, Spain; 3 Medical Genetics Unit, Central Laboratory, CHU Hassan II, Faculty of Medicine, Pharmacy and Dentistry, Sidi Mohamed Ben Abdellah University, Fez 30040, Morocco; b.elmakhzen@gmail.com (B.E.M.); laila.bouguenouch@yahoo.com (L.B.); 4 Institute of Sports Professions, Ibn Tofail University, Kenitra 14000, Morocco; s.elhrane@gmail.com; 5 Department of Pathology and Immunology, Faculty of Medicine, University of Geneva, 1211 Geneva, Switzerland; b.taib@yahoo.com; 6 Institute of Primary Care, University of Zurich, 8032 Zurich, Switzerland; katja@weiss.co.com (K.W.); beat.knechtle@hispeed.ch (B.K.); 7 Medbase St. Gallen Am Vadianplatz, 9000 St. Gallen, Switzerland; 8 M2S (Laboratoire Mouvement, Sport et Santé)—EA 1274, University of Rennes, 35000 Rennes, France; 9 Institut International des Sciences du Sport (2I2S), 35850 Irodouër, France

**Keywords:** cluster of differentiation 36, genetics, endurance athletes, team sports, athletic performance, exercise training

## Abstract

Previous studies have shown that variations in the *CD36* gene may affect phenotypes associated with fat metabolism as the CD36 protein facilitates the transport of fatty acids to the mitochondria for oxidation. However, no previous study has tested whether variations in the *CD36* gene are associated with sports performance. We investigated the genotypic and allelic distribution of the single-nucleotide polymorphism (SNP) rs1761667 in the *CD36* gene in elite Moroccan athletes (cyclists and hockey players) in comparison with healthy non-athletes of the same ethnic origin. Forty-three Moroccan elite male athletes (nineteen cyclists and twenty-four field hockey players) belonging to the national teams of their respective sports (athlete group) were compared to twenty-eight healthy, active, male university students (control group). Genotyping of the *CD36* rs1761667 (G>A) SNP was performed via polymerase chain reaction (PCR) and Sanger sequencing. A chi-square (χ^2^) test was used to assess the Hardy–Weinberg equilibrium (HWE) and to compare allele and genotype frequencies in the “athlete” and “control” groups. The genotypic distribution of the *CD36* rs1761667 polymorphism was similar in elite athletes (AA: 23.81, AG: 59.52, and GG: 16.67%) and controls (AA: 19.23, AG: 69.23, and GG: 11.54%; χ^2^ = 0.67, *p* = 0.71). However, the genotypic distribution of the *CD36* rs1761667 polymorphism was different between cyclists (AA: 0.00, AG: 72.22, and GG: 27.78%) and hockey players (AA: 41.67, AG: 50.00, and GG: 8.33%; χ^2^ = 10.69, *p* = 0.004). Specifically, the frequency of the AA genotype was significantly lower in cyclists than in hockey players (*p* = 0.02). In terms of allele frequency, a significant difference was found between cyclists versus field hockey players (χ^2^ = 7.72, *p* = 0.005). Additionally, there was a predominance of the recessive model in cyclists over field hockey players (OR: 0.00, 95% CI: 0.00–0.35, *p* = 0.002). Our study shows a significant difference between cyclists and field hockey players in terms of the genotypic and allelic frequency of the SNP rs1761667 of the *CD36* gene. This divergence suggests a probable association between genetic variations in the *CD36* gene and the type of sport in elite Moroccan athletes.

## 1. Introduction

Athletic performance is a multifaceted outcome influenced by the complex interaction of an athlete’s intrinsic and extrinsic factors. These include anthropometric parameters, physical and physiological capacities, the body’s adaptive response to training, nutritional considerations, psychological resilience, recovery capacity, susceptibility to injury, and the influence of environmental factors [1]. Furthermore, genetics can play a key role in determining an athlete’s level of performance [2]; genetic predisposition can make a significant contribution to an athlete’s abilities, influencing aspects such as muscle composition, oxygen transport capacity, and other physiological traits that have an impact on sporting success [3,4]. Previous research has identified a multitude of genes and polymorphisms, numbering over 250, which could potentially influence specific aspects of athletic performance [5]. These genetic factors may contribute to athletes’ inherent physiological capacities, shape their responses to physical training, and have an impact on their susceptibility to injury [5].

During exercise of low-to-moderate intensity, the main source of energy for skeletal muscle, particularly for type 1/oxidative fibers, comes from the oxidation of long-chain fatty acids (LCFAs) within the mitochondria [6]. However, the rate of oxidation of fatty acids during exercise is influenced by different variables: (i) the intensity and duration of physical effort; (ii) aerobic fitness; (iii) metabolic capacity; and (iv) the protein content necessary for the transport of fatty acids [6,7]. The capacity to oxidize high quantities of fatty acids can be attributed to mitochondrial adaptations to develop metabolic pathways to use fat as a fuel, as well as to mitochondrial density in the muscles concerned [8,9]. Previously, it was shown that mitochondrial density and maximal fat oxidation are greater in endurance athletes than in non-athletes [10]. These findings suggest that mitochondrial density and maximal fat oxidation may be associated with endurance athletes’ status and can influence athletic performance. In elite runners, the rate of fat oxidation is three times higher than in controls [11]. Additionally, and during high-intensity intermittent training (HIIT), fat oxidation can be up to 17 times greater in athletes than in untrained subjects, while carbohydrate oxidation may remain unchanged in both groups [12]. Interestingly, the passage of fatty acids across the plasma membrane is facilitated by various transport proteins [13].

Among these proteins is the cluster of differentiation 36 (CD36), an 88 kDa multifaceted glycoprotein encoded by the *CD36* gene. CD36 acts as the main transporter of long-chain fatty acids (LCFAs), facilitating their transport from adipose tissue to skeletal muscle cells and mitochondria [14,15,16]. CD36 is found in various tissues, including adipose tissue, blood cells, endothelium, heart, liver, and skeletal muscle [17,18]. Furthermore, CD36 is located on the external mitochondrial membrane and is involved in the activation, regulation, and transport of fatty acids [19]. During endurance exercise of low-to-moderate intensity, CD36 has been shown to play an essential role in lipolysis and the transport of fatty acids to the mitochondria for oxidation [6,20,21]. Additionally, a correlation has been observed between lipid oxidation during exercise and CD36 content [22,23] and between CD36 abundance and changes in maximal fat oxidation induced with training [24]. An improvement in muscle CD36 content and mitochondrial respiratory capacity was demonstrated by Warren et al. [25] after 8 to 16 weeks of endurance training. Furthermore, during acute endurance exercise, a gradual increase (30–60%) in CD36 protein content in mitochondria has been noted [26]. This increase was correlated with increased transport and the subsequent oxidation of LCFAs in skeletal muscle [26]. An improvement in mitochondrial content of CD36 has been also observed after seven sessions of HIIT [27]. This improvement was directly related to an increased ability to oxidize fat during HIIT sessions. However, in both active and inactive women, no significant increase in fat oxidation has been detected after HIIT [28,29]. Conversely, CD36 deficiency reduces aerobic exercise capacity due to decreased muscle uptake of fatty acids [16,30].

The *CD36* gene is located on chromosome 7—more precisely, on the long arm of band 11.2 (7q11.21) [31]—and it is composed of 15 exons coding for a single chain of 472 amino acids [32,33]. As a highly polymorphic gene, it contains several nucleotide variants in or out of the coding regions, which may decrease CD36 expression level, change the extracellular ligand-binding domain, or even cause CD36 deficiency [34]. To this end, several studies have examined the association of the *CD36* gene with various metabolic pathologies. CD36 deficiency has been shown to impair the myocardial uptake of LCFAs in humans [35]. Additionally, Tanaka et al. [36] suggested a potential link between CD36 deficiency and the development of hypertrophic cardiomyopathy. Clinical studies of CD36-deficient individuals indicate that its absence is associated with various metabolic abnormalities, including hyperlipidemia and insulin resistance [37,38]. Moreover, *CD36* polymorphisms have been associated with abnormal blood lipid levels [39], increased risk of coronary heart disease in diabetics [40], and increased risk of metabolic syndrome [41]. Among the variants of the *CD36* gene, there is a single-nucleotide polymorphism (SNP) known as rs1761667 that involves an A/G substitution [42]. This SNP gives rise to three possible genotypes (AA, AG, and GG) and associated phenotypes [42]. Specifically, individuals with the AA genotype of the *CD36* rs1761667 polymorphism have elevated levels of serum triglycerides and total cholesterol [42]. In addition, individuals carrying the A allele may undergo changes in lipid metabolism, which increase the risk of pathologies associated with atherosclerosis [43]. Subjects with the AA genotype of the rs1761667 polymorphism in the *CD36* gene have reduced expression of the functional CD36 protein. This decreased expression reduces the protein’s ability to absorb cholesterol efficiently, thus impairing its normal physiological function in cholesterol reuptake [44].

To the authors’ knowledge, no previous investigation has examined the potential association between *CD36* gene variations and elite athlete status, although this protein may be key for exercise performance phenotypes associated with fat oxidation (i.e., mainly for endurance athletes). For this reason, the main objective of this study was to study the genotypic and allelic distribution of SNP rs1761667 of the *CD36* gene in Moroccan elite athletes (road cyclists and field hockey players) and compare them with healthy non-athletic subjects. We selected these samples of athletes because road cyclists rely essentially on their aerobic metabolism to cover their energy needs, which are influenced by the intensity of the effort—generally low to moderate—and by the distance travelled (and cycling performance is intrinsically associated with aerobic variables such as maximal oxygen uptake and velocity at lactate threshold) [45,46]. On the other hand, although hockey players also rely on their aerobic energy system to meet the demands of the duration of the match [47], this is a high-intensity and intermittent team sport characterized by sudden sprints and bursts of intense action, which also leads to the activation of the anaerobic metabolism (most actions associated with success in field hockey are performed at high intensity) [47,48]. Overall, based on previous studies linking the AA genotype of *CD36* to certain pathologies, we hypothesized that the AA genotype will likely be less prevalent in athletes than in controls. Additionally, we hypothesized that the under-representation of the AA genotype would be more visible between athletes of an endurance sport (i.e., cycling, in which fat oxidation plays an important role) than in athletes of a high-intensity and intermittent sport (i.e., field hockey).

## 2. Materials and Methods

### 2.1. Participants

In this study, 43 elite Moroccan male athletes were recruited through contact with national sports federations. Among them, 19 participants were part of the national cycling team, including 2 cyclists who qualified for the 2024 Paris Olympic Games, 6 cyclists participating in the 2023 World Championships, and 2 cyclists who had triumphed in the Tour of Africa. The remaining 24 athletes belonged to the Moroccan national field hockey team, ranked 6th in Africa and 49th globally. We compared these athletes to a control group of 28 healthy male university students. These individuals were randomly selected from a larger group of students (totaling 69) at Ibn Tofail University in Kenitra, Morocco. All 71 participants underwent uniform anthropometric measurements and provided blood samples (4 mL), confidentially stored at −80 °C in the molecular biology laboratory of the University Hospital Center (CHU) in Fez, Morocco. This research has received the approval of the Research Ethics Committee of the Doctoral Center of Ibn Tofail University. This study strictly adhered to established protocols for biomedical research involving human subjects. All details regarding the procedures, potential benefits, and associated risks inherent in the study were carefully conveyed to all participants. The volunteers were properly informed of the importance of the study and voluntarily gave their written consent to participate in the experiment. The implementation of the procedures strictly followed the guidelines set out in the International Federation of Sports Medicine Consensus Statement on Genetic Information [49].

### 2.2. Genotyping

Genomic deoxyribonucleic acid (DNA) was extracted from leukocyte samples using a commercially available kit (MagPurix Blood DNA Extraction Kit) according to the manufacturer’s instructions. To determine DNA concentration, a nanodrop assessment was performed using a microspectrophotometer. Polymerase chain reaction (PCR) was performed to amplify the CD36 SNP rs1761667 (G>A) using an Applied Biosystems thermal cycler (VERITYTM). The primers used have already been described [50], namely, forward primer: 5′-CAAAATCACAATCTATTCAAGACCA-3′; and reverse primer: 5′-TTTTGGGAGAAATTCTGAAGAG-3′. The PCR procedure included an initial denaturation step at 94 °C for 5 min. This was followed by 30 amplification cycles, each consisting of denaturation at 94 °C for 30 s, annealing at 55 °C for 30 s, and extension at 72 °C for 30 s. A final extension step was carried out at 72 °C for 4 min. Additionally, PCR products were evaluated on a 2% agarose gel, accompanied by a size marker for molecular weight reference. Electrophoresis facilitated the separation of DNA fragments based on their size. Visualization under UV light, aided by ethidium bromide intercalation, provided information on the quality, quantity, and size distribution of the amplicons. Following the PCR technique, we performed enzymatic purification using ExoSAP-IT™ cleanup reagent (Thermo Fisher, Waltham, MA, USA) to remove and neutralize any remaining PCR residues. The DNA amplified was sequenced using the BigDye^®^ Terminator v. 3.1 Cycle Sequencing Kit by Applied Biosystems (Waltham, MA, USA). Finally, the sequences obtained were examined using Sequencing Analysis software, version 3.4.

### 2.3. Statistical Analysis

The conformity of genotype frequencies to Hardy–Weinberg equilibrium (HWE) was examined with the chi-square (χ^2^) test. This assessment involved a comparison between the observed genotype frequencies within each group and the expected genotype frequencies derived from the principles of HWE. For the categorical variables, the χ^2^ test was used to compare genotype and allele frequencies across all groups, including adjusted standardized residuals. Furthermore, and regarding the dominant (AA+AG vs. GG) and recessive (AA vs. AG+GG) models of the *CD36* rs1761667 polymorphism, in cyclists, field hockey players, all athletes, and controls groups, we also performed the χ^2^ test and calculated odds ratios (OR) with 95% confidence intervals (CI). The normality of continuous variables, particularly those associated with anthropometric data, was assessed via visual inspection using QQ-plots and via statistical analysis based on the D’Agostino–Pearson test. To assess the comparison between groups with respect to anthropometric data, one-way analysis of variance (ANOVA) was used when data were normally distributed. In cases where the data did not follow a normal distribution, Kruskal–Wallis’s test was applied. When a significant difference was detected between groups, post hoc multiple comparisons were performed. Specifically, Tukey’s test was used for parametric data, while Dunn’s test was used for nonparametric data. Consequently, the data were presented as means and standard deviations (SD) for parametric data and as medians and interquartile ranges (IQR, Q1–Q3) for non-parametric data. Finally, the threshold of statistical significance for all analyses was set at *p* < 0.050. Statistical analyses were carried out using GraphPad Prism 9.2.0, a software package supplied by GraphPad Software Inc. in San Diego, CA, USA.

## 3. Results

The anthropometric characteristics of all athletes and controls are shown in Table 1. Statistical analyses revealed significant differences in anthropometric data between groups, including age (controls vs. all athletes: *p* = 0.005; controls vs. cyclists: *p* < 0.0001; cyclists vs. field hockey: *p* < 0.001), body mass index (BMI) (controls vs. all athletes: *p* < 0.001; controls vs. cyclists: *p* = 0.003; controls vs. field hockey: *p* = 0.004), and body weight (controls vs. all athletes: *p* = 0.040). Nevertheless, all anthropometric data, except for age, exhibited similarities between cyclists and field hockey players.

**Figure 1:**
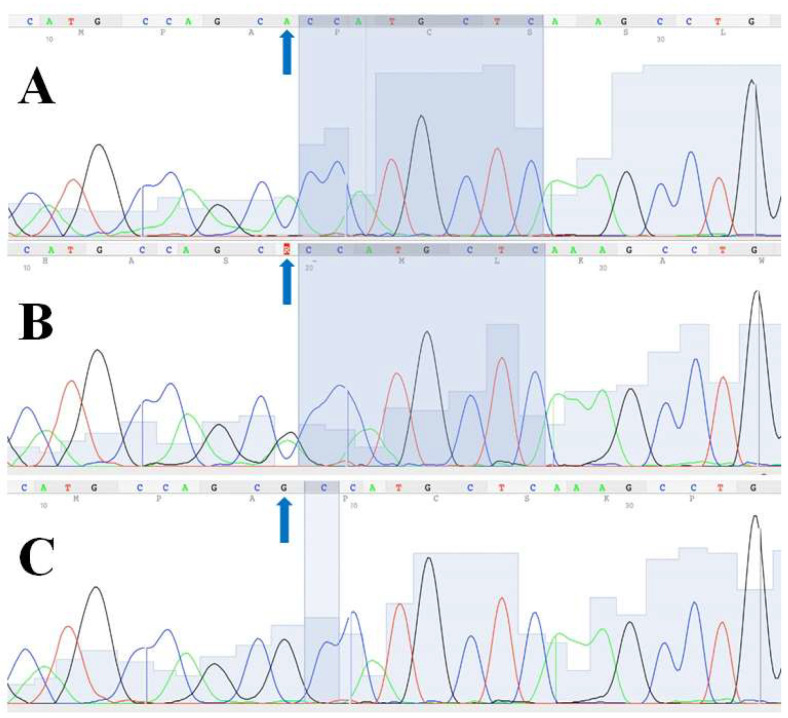
Fluorogram of the rs1761667 polymorphism from *CD36* gene sequencing: (**A**) homozygous for the A allele (AA); (**B**) heterozygous (AG); (**C**) homozygous for the G allele (GG). The arrows indicate the position of the CD36 rs1761667 polymorphism in three individuals with different genotypes.

The identification of the *CD36* SNP rs1761667 (G>A) was possible in all DNA samples (Figure 1) except for three samples. These samples pertained to one cyclist and two participants from the control group. The distribution of *CD36* genotypes did not differ significantly from HWE in any group. The distribution of genotypes and alleles for the *CD36* rs1761667 polymorphism is shown in Table 2. Interestingly, among the cyclists, the AG genotype was the most prevalent genotype (72.22%), while none of the cyclists had the AA genotype. Consequently, the frequency of the A allele (36.11%) was lower than that of the G allele (63.89%) in the group of cyclists. In field hockey players, the AG genotype was also the most prevalent (AG: 50% > GG: 41.67% > AA: 8.33%), but in this case, the frequency of the A allele (66.67%) was higher when compared to the G allele (33.33%) (which is the opposite for cyclists). Concerning the all-athlete group, we noted marked predominance of the AG genotype compared to the AA and GG genotypes (AG: 59.52% > AA: 23.81% > GG: 16.67%). Additionally, the frequency of the A allele (53.57%) was slightly higher than that of the G allele (46.43%). Finally, in the control group, the AG genotype was also superior to the AA and GG genotypes (AG: 69.23% > AA: 19.23% > GG: 11.54%), and the frequency of the A allele (53.85%) was a little higher than that of the G allele (46.15%).

Regarding the comparison of *CD36* genotypes between groups (Table 3), our analysis did not reveal statistically significant differences in the following comparisons: (i) controls vs. all athletes (χ^2^ = 0.67, *p* = 0.71); (ii) controls vs. cyclists (χ^2^ = 5.01, *p* = 0.08); and (iii) controls vs. field hockey players (χ^2^ = 2.99, *p* = 0.22). On the other hand, a significant difference was observed between cyclists and field hockey (χ^2^ = 10.69, *p* = 0.004). Specifically, the frequency of the AA genotype was significantly lower among cyclists than in hockey players (*p* = 0.02), with no differences between athletes in the other two genotypes. Furthermore, and in terms of allele frequency comparison (Table 4), a significant difference was found when comparing cyclists vs. field hockey (χ^2^ = 7.72, *p* = 0.005). Conversely, our analysis showed no significant differences in the comparison between controls and all athletes (χ^2^ = 0.00, *p* = 0.97), controls and cyclists (χ^2^ = 2.69, *p* = 0.10), and controls and field hockey athletes (χ^2^ = 1.70, *p* = 0.19).

In the final analysis, for the dominant model (AA+AG vs. GG) of *CD36* rs1761667 polymorphism (Table 5), no significant difference was shown in the following comparisons: (i) controls vs. all athletes (OR: 0.65, 95% CI: 0.17–2.84, *p* = 0.73); (ii) controls vs. cyclists (OR: 0.33, 95% CI: 0.08–1.70, *p* = 0.24); (iii) controls vs. field hockey players (OR: 1.43, 95% CI: 0.26–8.62, *p* = 0.99); and (iv) cyclists vs. field hockey players (OR: 0.23, 95% CI: 0.04–1.46, *p* = 0.11). Regarding the recessive model (AA vs. AG+GG) of the *CD36* gene (Table 5), no statistically significant differences were observed in the following comparisons: (i) controls vs. all athletes (OR: 1.31, 95% CI: 0.38–3.93, *p* = 0.76); (ii) controls vs. cyclists (OR: 0.00, 95% CI: 0.00–0.95, *p* = 0.06); and (iii) controls vs. field hockey players (OR: 3.00, 95% CI: 0.90–9.97, *p* = 0.12). However, a statistically significant result was detected when comparing the recessive model of cyclists and field hockey players (OR: 0.00, 95% CI: 0.00–0.35, *p* = 0.002).

## 4. Discussion

During aerobic exercise of low-to-moderate intensity, fatty acids are the most important substrates for energy production in skeletal muscles [51]. In addition, prolonged and intense exercise and insufficient carbohydrate intake can restrict muscle glycogen reserves and reduce athletic performance [52,53] as the production of energy is lower when it relies principally on fat rather than on carbohydrate oxidation. Consequently, aerobic physical training increases the expression of proteins involved in the oxidative metabolism in human skeletal muscle [54]. Among these proteins is the CD36, which plays a crucial role in the transport of fatty acids from adipose tissue to muscle mitochondria and facilitates fat metabolism [55,56]. The main objective of this study was to examine the distribution of genotypic and allelic variations of the *CD36* rs1761667 polymorphism in high-level athletes (cyclists and field hockey athletes) in Morocco in comparison with those of non-athletes (healthy subjects) of the same ethnic origin. With regard to the main results of this study, there was no significant difference between athletes and controls in terms of the distribution of the rs1761667 polymorphism in the *CD36* gene. However, a significant difference was found between cyclists and field hockey players in terms of genotypes (*p* = 0.004) and alleles (0.005) of the *CD36* rs1761667 polymorphism. Specifically, there was a higher frequency of the G allele in cyclists with respect to field hockey players, while the AA genotype was underrepresented. Additionally, the recessive model (AA vs. AG+GG) of the *CD36* rs1761667 polymorphism revealed a significant difference between cyclists and field hockey players (*p* = 0.002). This divergence suggests a probable association between genetic variations in the *CD36* gene and the type of sport in elite Moroccan athletes. Collectively, it seems that the AA genotype may hinder the likelihood of becoming an elite cyclist as no participant had this genotype. On the other hand, possessing the G allele may facilitate reaching the status of elite cyclists. This is a hypothesis that require further confirmatory studies but agrees with the “negative” phenotypes for fat metabolism linked to the AA genotype of the *CD36* rs1761667 polymorphism, which may also be “negative” for an endurance sport like cycling in which reliance on fat oxidation for the obtaining of energy is crucial.

To our knowledge, our study is the first to examine the association between the rs1761667 polymorphism of the *CD36* gene and the status of elite athletes. Therefore, we did not find similar data on the rs1761667 polymorphism in athletes with which to compare our results. Some studies specifically investigate CD36 protein expression in response to physical exercise and fat oxidation. For example, Bruce et al. [57] showed no significant difference in CD36 content between type 2 diabetes, young, and trained groups. In addition, an increase in CD36 was demonstrated after endurance [58,59] and short-term exercise [60] in endurance-trained subjects. Furthermore, a correlation between peak fat oxidation and CD36 content during exercise was found in endurance athletes [24]. Specifically, Fujii et al. [61] show a link between the AA rs1761667 genotype of the *CD36* gene and a high level of total fat. Moreover, in our recently published study [21], we found a remarkable association between CD36 and lipolysis of adipose tissue during physical training. In addition, our research identified a correlation between peak fat oxidation and CD36 expression [21]. These results highlight the relationship between CD36 expression, and the physiological responses associated with exercise-induced adipose tissue metabolism.

Overall, and in light of previous investigations, the *CD36* gene is frequently studied in patients in order to assess its association with certain pathologies. The AA genotype of SNP rs1761667 in the *CD36* gene showed a higher prevalence in the obese group than in the normal-weight group [62]. Additionally, the AA genotype was associated with higher levels of serum triglycerides and total cholesterol [42]. In the Jordanian population, Hatmal et al. [63] found no association between the *CD36* gene and the presence of type 2 diabetes or dyslipidemia. A correlation between the *CD36* rs1761667 polymorphism and susceptibility to hypertension has been demonstrated in the Iranian population [64]. In a similar context, there is a suggestive hypothesis that a decrease in CD36 expression in renal cells could be linked to hypertension [65]. Conversely, it has been suggested that the AA genotype of rs1761667 in the *CD36* gene is associated with a lower risk of hypertension in a Japanese population [61]. Finally, the function of the *CD36* gene in the transport and oxidation of fatty acids during physical exercise has been confirmed in the majority of studies [21,61,66]. These results may suggest that carrying a genotype that increases CD36 activity may have a differential effect on the physiological capacity and athletic performance of athletes. Furthermore, an association was demonstrated between the rs1761667 SNP in the *CD36* gene and a reduction in CD36 protein expression [67]. Specifically, and according to Daoudi et al. [68], a higher frequency of AA and AG genotypes was observed in obese adolescents than the GG genotype. On the other hand, our results reveal a dominance of the G allele over the A allele in cyclists, while in hockey players, the A allele predominates compared to the G allele. These results may indicate a possible association between the rs1761667 polymorphism of the *CD36* gene and athletic performance in elite Moroccan athletes. Specifically, it appears that individuals carrying the G or A alleles have an advantage in some sports, but not necessarily in others. This suggests a nuanced relationship between genetic variations in the *CD36* gene and sport-specific advantages.

In our study, we investigated the genotypic distribution of the *CD36* rs1761667 SNP in Moroccan elite athletes and non-athletes. Although our study is the first to investigate this gene in elite athletes, it has some limitations. First, the inability to compare results due to the lack of similar data in existing studies. Additionally, the relatively small sample size (n = 43) may be considered a limiting factor. Consequently, further studies are imperative to validate and substantiate the potential impact of the variants in the *CD36* gene on elite athletes. Furthermore, our data are limited to frequency measurements, and there is a lack of information on performance phenotypes such as VO_2_ peak, maximal fat oxidation rates during exercise or blood lipid profiles.

## 5. Conclusions

In conclusion, our results indicate a significant difference in the genotypic and allelic distribution of the *CD36* rs1761667 SNP between cyclists and field hockey players. Cyclists have a higher prevalence of the G allele when compared to field hockey players, while no cyclists have the AA genotype. This divergence suggests a probable association between genetic variations in the *CD36* rs1761667 SNP and the type of sport in elite Moroccan athletes. However, this is a pilot study, and there is a need of further research on this topic to confirm our main findings.

## Figures and Tables

**Table 1:** Anthropometric data of elite Moroccan athletes of individual (cycling) and team (field hockey) sports vs. a control group of non-athletes.

	Cyclists	Field Hockey Players	All Athletes	Controls	*p*-Value	Controls vs. All Athletes	Cyclists vs. Field Hockey Players
Age (year)	23.58 ± 3.76	18.5 (18–20.75)	20 (18–23)	18.71 ± 0.76	<0.0001	0.005	<0.001
Weight (kg)	64.37 ± 4.90	64.25 ± 7.22	64.30 ± 6.23	72 (62–80.50)	0.03	0.04	>0.999
Height (m)	1.77 ± 0.05	1.76 ± 0.05	1.77 ± 0.05	1.76 ± 0.08	0.78	0.96	0.81
BMI (kg/m^2^)	20.50 ± 1.90	20.75 ± 2.00	20.64 ± 1.93	23.04 ± 3.43	<0.001	<0.001	0.98

Values are presented as means and standard deviations (SD, normally distributed variables) or medians and interquartile ranges (IQR, not normally distributed variables). BMI: body mass index.

**Table 2:** Genotypic and allelic frequencies of the *CD36* rs1761667 polymorphism in elite Moroccan athletes of individual (cycling) and team (field hockey) sports versus a control group of non-athletes.

Variables		Cyclists	Field Hockey Players	All Athletes	Controls
Sample size		18	24	42	26
Genotype distribution, n (%)	AA	0 (0.00%)	10 (41.67%)	10 (23.81%)	5 (19.23%)
	AG	13 (72.22%)	12 (50.00%)	25 (59.52%)	18 (69.23%)
	GG	5 (27.78%)	2 (8.33%)	7 (16.67%)	3 (11.54%)
Allele distribution, n (%)	A	13 (36.11%)	32 (66.67%)	45 (53.57%)	28 (53.85%)
	G	23 (63.89%)	16 (33.33%)	39 (46.43%)	24 (46.15%)
HWE *p*-value		0.06	0.83	0.44	0.13

HWE: Hardy–Weinberg equilibrium.

**Table 3:** Comparison of the genotypic distribution of the *CD36* rs1761667 polymorphism (AA vs. GA vs. GG) in elite Moroccan athletes of individual (cycling) and team (field hockey) sports versus a control group of non-athletes.

Groups	χ^2^	df	*p*-Value
Controls vs. All athletes	0.67	2	0.71
Controls vs. Cyclists	5.01	2	0.08
Controls vs. Field hockey players	2.99	2	0.22
Cyclists vs. Field hockey players	10.69	2	0.004

df: Degrees of freedom.

**Table 4:** Comparison of the *CD36* rs1761667 polymorphism alleles (G vs. A) in elite Moroccan athletes of individual (cycling) and team (field hockey) sports versus a control group of non-athletes.

Groups	χ^2^	df	*p*-Value
Controls vs. All athletes	0.00	1	0.97
Controls vs. Cyclists	2.69	1	0.10
Controls vs. Field hockey players	1.70	1	0.19
Cyclists vs. Field hockey players	7.72	1	0.005

df: Degrees of freedom.

**Table 5:** Odds ratios [95% confidence intervals] for the predominance of dominant and recessive models of the *CD36* rs1761667 polymorphism in elite Moroccan athletes of individual (cycling) and team (field hockey) sports versus a control group of non-athletes.

Groups	Dominant (AA+AG vs. GG)	*p* Value	Recessive (AA vs. AG+GG)	*p*-Value
Controls vs. All athletes	0.65 [0.17–2.84]	0.73	1.31 [0.38–3.93]	0.76
Controls vs. Cyclists	0.33 [0.08–1.70]	0.24	0.00 [0.00–0.95]	0.06
Controls vs. Field hockey players	1.43 [0.26–8.62]	0.99	3.00 [0.90–9.97]	0.12
Cyclists vs. Field hockey players	0.23 [0.04–1.46]	0.11	0.00 [0.00–0.35]	0.002

## Data Availability

The original contributions presented in the study are included in the article. Further inquiries can be directed to the corresponding authors.
